# The Remarkable I_2_O_3_ Molecule: A New View from Theory

**DOI:** 10.1021/acs.jpca.5c04243

**Published:** 2025-08-04

**Authors:** Carson L. Tang, Justin M. Turney, Henry F. Schaefer

**Affiliations:** Center for Computational Quantum Chemistry, Department of Chemistry, 1355 University of Georgia, Athens, Georgia 30602, United States

## Abstract

Atmospheric iodine chemistry has garnered increasing attention as a result of increased iodine emissions. A key subset of this chemistry involves iodine oxides (I_2_O_2–5_), which serve as precursors to particle formation. Among these, I_2_O_3_ is the simplest iodine oxide involved in particle formation, but it has remained undetected in the atmosphere. Previous theoretical studies have characterized this peculiar molecule, primarily using energies to refine geometries obtained at low levels of theory. Due to the reemerging interest in I_2_O_3_, this study presents geometries optimized at the CCSD­(T)/aug-cc-pwCVTZ-PP level of theory—marking the first instance, to the best of our knowledge, where this system has been studied exclusively with CCSD­(T). Harmonic vibrational frequencies were computed at the same level of theory. Final energetics were obtained using the very high level CCSDT­(Q) method with basis sets up to quintuple-zeta cardinality (aug-cc-pwCV5Z-PP) and extrapolated to the CBS limit to yield CCSDT­(Q)/CBS//CCSD­(T)/aug-cc-pwCVTZ-PP energies. These energies include harmonic zero-point vibrational energy corrections and scalar relativistic energy corrections. Additionally, this study discovers new isomers along the I_2_O_3_ potential energy surface, a novel contribution to the field. The performance of different computational methods and DFT functionals commonly used in atmospheric chemistry is also assessed relative to high-level theoretical methods.

## Graphical abstract

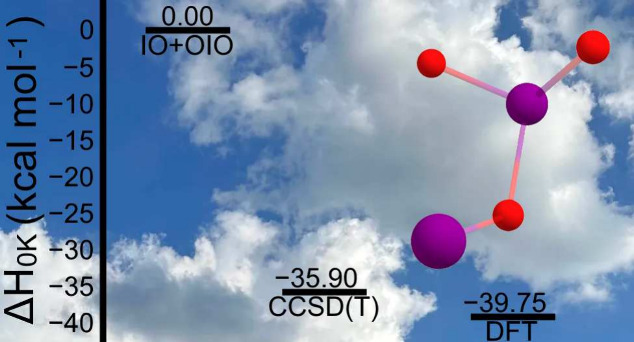

## Introduction

Aerosols in the atmosphere have been shown to impact the Earth–atmosphere system, showing effects on air quality, human health, weather, and climate.^1^ Aerosols also act as cloud condensation nuclei (CCN), which have been hypothesized to accelerate the melting of sea ice.^1,2^ Given their significant role in the atmosphere, understanding how these particles are formed is crucial. A major source of these particles is the result of new particle formation (NPF), which accounts for approximately 50% of CCN in the Earth’s atmosphere. NPF begins with the formation of clusters in the gas phase, which grow in size as they collide with surrounding clusters.^1^ The most prevalent nucleating species has historically been sulfuric acid, due to its low vapor pressure under typical atmospheric conditions, in addition to its ability to form hydrogen bonds with many important compounds in the atmosphere.^1,3^ Additional nucleating precursors reported in the literature include: ammonia/amines, highly oxygenated molecules, organic compounds, iodine oxides, and iodine oxyacids.^1,4–7^ However, uncertainties remain regarding the mechanisms of NPF and the chemical species involved. Available NPF data come from field measurements, laboratory experiments, and theoretical studies, but these results are often conflicting. While theoretical work in this field has been relatively rare, it has been advocated as a valuable complement to field measurements and experimental results.^1^ Iodine oxyacids, such as HIO_3_ and HIO_2_, have been proposed to be the main drivers in NPF.^5–7^ One possible pathway for the formation of these oxyacids is the reaction of iodine oxides with water.^8^ Therefore, understanding the properties and behavior of iodine oxides is just as important as studying the iodine oxyacids themselves.

Iodine oxides are the most obscure of the nucleating species mentioned above. The atmospheric chemistry of iodine has gained increasing importance, as evidenced by the abundance of references to the review by Saiz-Lopez et al. in 2011.^9^ Atmospheric iodine-containing species in general have risen in concentrations since the 1950s, a result of rising ozone (O_3_) levels from both natural and anthropogenic sources.^3,7,10–13^ Seaweeds and algae in coastal waters emit diatomic iodine (I_2_) and organoiodine compounds (e.g., CH_3_I) into the atmosphere, photochemically producing the iodine radical (I^•^).^9,10^ Ozone in the atmosphere then reacts with I^•^ to produce iodine monoxide (IO) and iodine dioxide (IO_2_).^9,10^ As described in the influential review by Saiz-Lopez et al., higher-order iodine oxides (I_2_O_2–5_) are produced from a series of self-reactions between IO and OIO.^9^ The simplest iodine oxide that directly leads to particle formation is I_2_O_3_, a species frequently noted but not well characterized.^2–4,10,14–93^

Kaltsoyannis and Plane performed early quantum chemical calculations on I_2_O_3_ as part of a study of important iodine containing compounds in the atmosphere in 2008.^89^ At the CCSD­(T)/aug-cc-pVTZ//B3LYP/aug-cc-pVTZ level of theory, they concluded that I_2_O_3_ is a very stable molecule with C_1_ symmetry.^89^ They also reported harmonic vibrational frequencies, but did not include the intensities or symmetries of the vibrational modes. In 2013, a combined experimental (Gómez Martín et al.) and theoretical (Gálvez et al.) study provided the first direct experimental evidence of I_2_O_3_ in the gas phase.^90,91^ The theoretical portion of this study reported CCSD­(T) energies computed on MP2 or B3LYP geometries.^90^ However, Gálvez et al. only report bond lengths of I_2_O_3_, omitting bond angles and harmonic frequencies.^90^ Gómez Martín et al. conducted another combined experimental and theoretical study in 2020 to explain atmospheric particle formation through clustering of iodine oxides.^92^ This study included a potential energy surface for the reaction of I_2_O_3_ with water, calculated at the CCSD­(T)/aug-cc-pVTZ+LAN2LDZ//M06-2X/aug-cc-pVDZ+LANL2DZ level of theory.^92^ More recently, in the latter half of 2024, Ning et al. presented a study on the heterogeneous chemistry of I_2_O_3_, including molecular dynamics simulations and quantum chemical calculations of I_2_O_3_ at the DLPNO-CCSD­(T)/aug-cc-pVTZ­(-PP)//M06-2X/aug-cc-pVTZ­(-PP) level of theory.^93^ Engsvang, Wu, and Elm recently conducted a computational benchmark of iodine oxyacid and oxide dimers.^94^ While this benchmark only included I_2_O_4_ and I_2_O_5_, the title suggests a future benchmark that may include I_2_O_3_. They found that optimizing structures at the ωB97X-D3BJ/aug-cc-pVTZ-PP or M06-2X/aug-cc-pVTZ-PP level of theory achieved the best thermal contribution to the binding free energy.^94^ Electronic energy corrections were computed using the ZORA-DLPNO-CCSD­(T_0_) method with the SARC-ZORA-TZVPP basis set for iodine and the ma-ZORA-def2-TZVPP basis set for noniodine atoms.^94^

Despite experimental observations, the structure and infrared spectrum of I_2_O_3_ have yet to be reported. Field measurements have thus far failed to detect I_2_O_3_ in the atmosphere. This may be due to I_2_O_3_ being considered a “dead end” in the current understanding of iodine chemistry in the atmosphere.^93^ However, recent studies have shown continued interest in this peculiar system and atmospheric iodine chemistry in general.^4,10,92–94^ For example, recent work by Francisco and co-workers highlights that I_2_O_3_ plays a critical role in the ocean–atmosphere iodine cycle.^93^

Previous theoretical works report CCSD­(T) or DLPNO-CCSD­(T) energies on geometries optimized at lower levels of theory (e.g., DFT).^89,90,92,93^ In the present study, I_2_O_3_ has been fully optimized using the CCSD­(T) method. With these structures, much higher levels of theory have been applied to I_2_O_3_. The results will be compared with other *ab initio* methods and various DFT functionals commonly seen in atmospheric chemistry. Additional isomers on the potential energy surface are also presented. To the best of our knowledge, this is the first study to optimize I_2_O_3_ with the CCSD­(T) method and to present additional minima along the PES, regardless of the level of theory.

## Computational Methods

Here, computations were performed using a variety of post-Hartree–Fock methods and, for comparison, DFT functionals. Geometry optimization and harmonic vibrational frequency evaluations for the I_2_O_3_ isomer lowest in energy were carried out with the MP2, CCSD, and CCSD­(T) methods.^95–108^ Functionals frequently used in atmospheric chemistry were also employed to assess their performance in predicting geometric parameters and vibrational frequencies against the gold standard CCSD­(T).^3^ These functionals include M06-2X, PW91, ωB97X-D, and the familiar B3LYP.^109–112^ In the present research, six different basis set combinations were used to assess basis set dependence as well. The first combination employed the cc-pVXZ (X = D, T) basis sets for oxygen atoms and the cc-pVXZ-PP basis sets for iodine atoms, and will be abbreviated as XZ.^113–119^ The second combination used the aug-cc-pVXZ basis sets for oxygen atoms and aug-cc-pVXZ-PP basis sets for iodine atoms, abbreviated as AVXZ.^113–116,120,121^ The third combination still used the aug-cc-pVXZ basis sets for oxygen atoms but utilized the larger aug-cc-pwCVXZ-PP basis sets for iodine atoms, abbreviated as ACVXZ.^113–116,120,121^ This combination was suggested by Peterson, who noted that additional tight functions are essential for determining accurate structures and energetics for IO and OIO.^122^ The structures and harmonic vibrational frequencies of the reactants that form I_2_O_3_, IO and OIO, in addition to the higher energy I_2_O_3_ isomers, were also computed but only at the CCSD­(T)/ACVTZ level of theory. For the basis sets used within this study, the -PP appended to the end of a basis set refers to a relativistic effective core potential (ECP) that replaces the 28-electron core of the iodine atom. This is described by the noble gas configuration [Ar]­4s^2^3d^8^ and used to reduce the number of explicitly correlated electrons from 53 to 25.

Post-Hartree–Fock calculations for all the I_2_O_3_ isomers were computed with CFOUR version 2.1 with the NCC module.^123^ Post-Hartree–Fock calculations for IO and OIO were computed with the VCC module of CFOUR, using an ROHF reference function. Unless otherwise noted, all post-Hartree–Fock computations were obtained from CFOUR. All DFT calculations were computed using Psi4 version 1.9 using an ultrafine grid.^124–127^ In all cases, geometries were converged to an RMS gradient below 10^–8^ hartree bohr^–1^. The Hartree–Fock and coupled cluster amplitude equations were also converged to 10^–10^ hartrees. Harmonic vibrational frequencies were obtained by finite differences of analytic gradients, following each geometry optimization to confirm that the final structure was a minimum along the potential energy surface. Anharmonic frequencies were computed for the global minimum with VPT2 using CFOUR.^128^

Electronic energies for each stationary point along the potential energy surface were computed using the focal point approach at the geometries obtained at the highest level of theory, CCSD­(T)/ACVTZ.^129–132^ Basis sets up to quintuple-zeta cardinality were used (O: aug-cc-pVXZ; I: aug-cc-pwCVXZ-PP, X = D, T, Q, 5) for Hartree–Fock, MP2, CCSD, and CCSD­(T) energies, computed with the 2022 version of Molpro for the I_2_O_3_ isomers, IO, and OIO.^133–135^ The energies obtained were extrapolated to the complete basis set limit using a three-point fitting equation for the Hartree–Fock energies and a two-point fitting equation for the correlation energies:

1 $$E_{HF}=A+Be^{-CX}$$

2 $$E_{corr}=A+BX^{-3}$$

Additional corrections were then appended to the resulting net energies to determine relative energies at 0 K. These corrections include a CCSDT correction (δ_T_), a CCSDT­(Q) correction (δ_(Q)_), a harmonic zero-point vibrational energy correction (δ_ZPVE_), a scalar relativistic energy correction (δ_rel_), and a spin-orbit correction (δ_SO_).^136–141^

The CCSDT corrections were obtained at the CCSDT/ACVDZ level of theory using CFOUR for the I_2_O_3_ isomers and MRCC for IO and OIO.^142,143^ The CCSDT­(Q) corrections were obtained at the CCSDT­(Q)/DZ level of theory using MRCC for all minima. For the open-shell species, the CCSDT­(Q)/B energy was used.^144^ The zero-point vibrational energy corrections were obtained from the harmonic vibrational frequency computations. Relativistic energy corrections were computed at the scalar level, without the inclusion of spin–orbit coupling. These energy corrections were computed with the X2C1E treatment in CFOUR, using the seg-cc-pVTZ-X2C basis set for oxygen atoms and the seg-cc-pwCVTZ-X2C basis set for iodine atoms.^145–152^ Spin–orbit effects were treated with the internally contracted multireference configuration method in Molpro with a cc-pVTZ-DK basis set on all atoms. The active space configurations were defined as (7,4) for IO, full valence for OIO, and (6,6) for I_2_O_3_. By appending these additional corrections to the net energies obtained from the focal point approach, relative energies are given by the following:

3 $$\Delta_{f}H_{0}=\Delta E_{CCSD\left(T\right)/CBS}+\delta_{T}+\delta_{\left(Q\right)}+\delta_{ZPVE}+\delta_{\mathrm{rel}}+\delta_{SO}$$

## Results and Discussion

### Geometries

Shown in Figure 1 are the qualitative geometries of the IO, OIO, and I_2_O_3_ structures obtained at the CCSD­(T)/ACVTZ level of theory. The isomer labeled as M1, located on the bottom left of Figure 1, is the global minimum that has been considered in previous reports. To the best of our knowledge, the additional I_2_O_3_ isomers (M2–M5) have not been reported until now. These additional structures were identified by examining the potential energy surfaces of their corresponding lighter halogen analogs (Y_2_O_3_, Y = F, Cl, Br).^153–158^ Notably, isomers M2 and M5 are enantiomers of M2′ and M5′, respectively. They share the same bond lengths, same vibrational modes, and are isoenergetic with each other. The present study focuses primarily on the M1 isomer, which is shown isolated in Figure 2. Geometric parameters for IO, OIO, and the additional I_2_O_3_ structures are shown in the Supporting Information.

**Figure 1:**
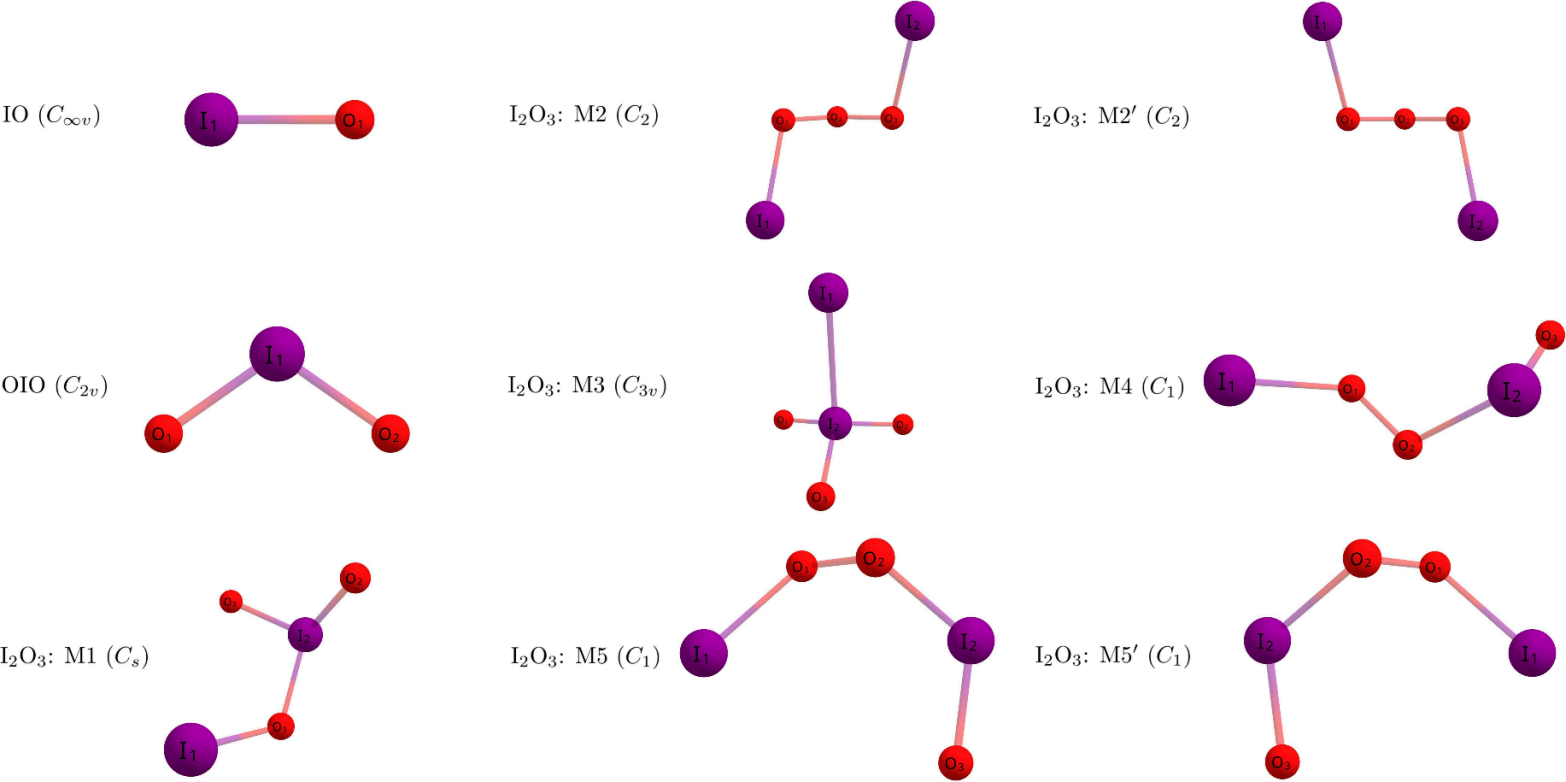
Qualitative geometries of IO, OIO, and I_2_O_3_ structures obtained at the CCSD­(T)/aug-cc-pwCVTZ-PP level of theory.

**Figure 2:**
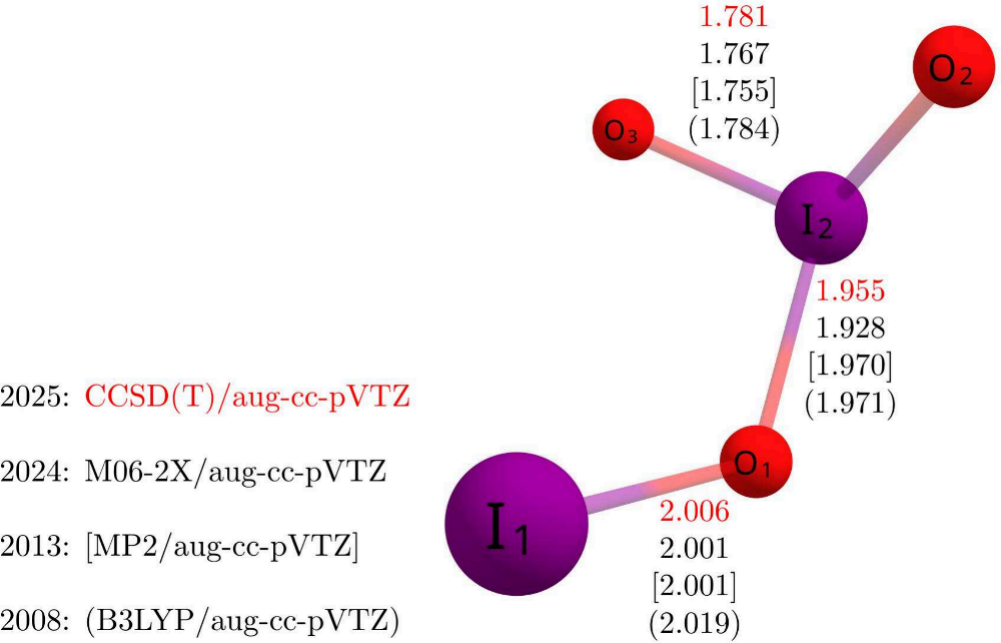
Predicted values of the I_2_O_3_ bond lengths (Å) at the CCSD­(T)/aug-cc-pVTZ-PP level of theory (shown in red) compared to values previously reported in the literature from different methods.^89,90,93^ Point group: *C*_*s*_. See the text for different ECPs used.

In Figure 2, bond lengths of the M1 isomer obtained in this study at the CCSD­(T)/AVTZ level of theory are displayed in red print. For comparison, previously reported bond lengths obtained using M06-2X, MP2, and B3LYP, all with the same aug-cc-pVTZ basis sets, are shown in black with square brackets and parentheses.^89,90,92^ All bond lengths are reported in angstroms. While there is some variation between the four sets of values shown, none match the CCSD­(T) results closely. However, certain values, such as the I_2_–O_3_ bond length, differ by only 0.003 Å. These variations may in part be attributed to differences in the effective core potentials (ECPs) used for iodine in the various studies. For instance, Kaltsoyannis and Plane (2008) used Glukhovstev’s all-electron iodine basis set, while Gálvez et al. (2013) employed an ECP replacing the 46-electron core of iodine. The present study uses the same Peterson ECP as Francisco and co-workers, which replaces the 28-electron core of iodine.^121^ Benchmark studies of ECPs have demonstrated that outer-core electrons play an important role in accurately determining bond lengths and related properties.^121^

To provide a consistent comparison, our study presents bond lengths, bond angles, and dihedral angles computed using identical basis sets and ECPs across all methods and DFT functionals. The results are tabulated in the Supporting Information, showing raw geometric values and values relative to CCSD­(T) results since there are currently no experimental data. These tables include data from seven methods: B3LYP, M06-2X, PW91, ωB97X-D, MP2, CCSD, and CCSD­(T). Each method was used with six basis set combinations: DZ, AVDZ, ACVDZ, TZ, AVTZ, and ACVTZ, yielding a total of 42 levels of theory.

For this study, small deviations were defined as bond length differences less than 0.01 Å and bond angle or dihedral deviations of less than 1°. Based on our results, B3LYP and MP2 performed the best while CCSD and PW91 performed the worst in predicting geometries compared to CCSD­(T). Specifically, B3LYP and MP2 yielded 27/42 (64.3%) and 26/42 (61.9%) values that had smaller deviations, respectively. The CCSD and PW91 methods, yielded 17/42 (40.5%) and 16/42 (38.1%) values with small deviations, respectively. From our results, mean absolute errors (MAE), root-mean-square errors (RMSE), and maximum errors were determined for bond lengths and bond angles to see how each method performed in comparison to CCSD­(T). These values are shown in Table 1.

**Table 1:** Performance of Different Methods in Predicting Equilibrium Geometries Relative to CCSD­(T) Shown by Mean Absolute Error, Root-Mean-Square Error, and Maximum Error

	Bond Lengths (Å)			Bond Angles (deg)		
	MAE	RMSE	Max. Error	MAE	RMSE	Max. Error
MP2	0.016	0.019	0.035	0.8	1.2	2.6
CCSD	0.025	0.028	0.048	0.8	1.2	2.8
B3LYP	0.012	0.015	0.034	1.3	2.0	4.5
M06-2X	0.027	0.031	0.058	0.9	1.2	3.0
PW91	0.032	0.041	0.080	0.8	1.0	1.7
ωB97X-D	0.015	0.018	0.034	1.6	2.5	6.1

Based on the values in Table 1, B3LYP is the method that predicts bond lengths closest to CCSD­(T) while PW91 is the method that predicts bond angles and dihedrals closest to CCSD­(T). Interestingly, while PW91 is able to predict bond angles, it performs the worst in predicting bond lengths. In predicting equilibrium geometries as a whole, it would seem MP2 is the method that is able to predict geometries closest to CCSD­(T) quality. As for DFT functionals, B3LYP is likely the best choice, having the smallest errors in bond lengths and reasonable errors for bond angles.

### Energetics

To determine relative energies, I_2_O_3_ is described relative to the reactants by the focal point table shown by Table 2.^129–132^ The energy is still changing by the time CCSD­(T) is introduced, with a subsequent change of 1.29 kcal mol^–1^. This raises a concern for multireference character in the system. Inspection of diagnostics such as T_1_ and T_2_ amplitudes however, presented no major multireference character. Higher order methods such as CCSDT and CCSDT­(Q) were used to address this concern. The full CCSDT correction is still high with a change of 1.05 kcal mol^–1^. The CCSDT­(Q) correction is within chemical accuracy (1 kcal mol^–1^), but is still not as converged as one would hope with CCSDT­(Q), with a change of 0.61 kcal mol^–1^. It would seem that even higher order methods such as CCSDTQ or CCSDTQ­(P) may be needed to precisely converge the energy of this system. The total relativistic correction of 8.20 kcal mol^–1^ is the largest correction. However, this large change does not seem too alarming as previous studies have stated that inclusion of relativistic effects may cause the relative energy to increase.^89,159^ The focal point tables of the other I_2_O_3_ isomers can be found in the Supporting Information. Figure 3 shows the energies of all I_2_O_3_ isomers relative to the reactants.

**Table 2:** Incremental Focal Point Table for the M1 Isomer Relative to the Reactants, IO and OIO; The Final Relative Energy Is Obtained as Δ_f_ *H*_0_ = Δ*E*_CCSD(T)/CBS_ + δ_T_ + δ_(Q)_ + δ_ZPVE_ + δ_rel_ + δ_SO_

Basis Set	RHF	+δ_MP2_	+δ_CCSD_	+δ_(T)_	Net
DZ	–20.11	–22.72	+9.04	–1.29	–35.07
TZ	–26.93	–25.91	+10.32	–1.40	–43.92
QZ	–27.78	–26.47	+10.13	–1.44	–45.56
5Z	–27.82	–26.89	+10.09	–1.47	–46.09
CBS	[−27.78]	[−26.87]	[+9.99]	[−1.47]	[−46.13]
Δ_f_ *H*_0_ = −46.13 + 1.05 – 0.61 + 1.59 + 7.20 + 1.00 = −35.90 ± 0.08 kcal mol^–1^					

**Figure 3:**
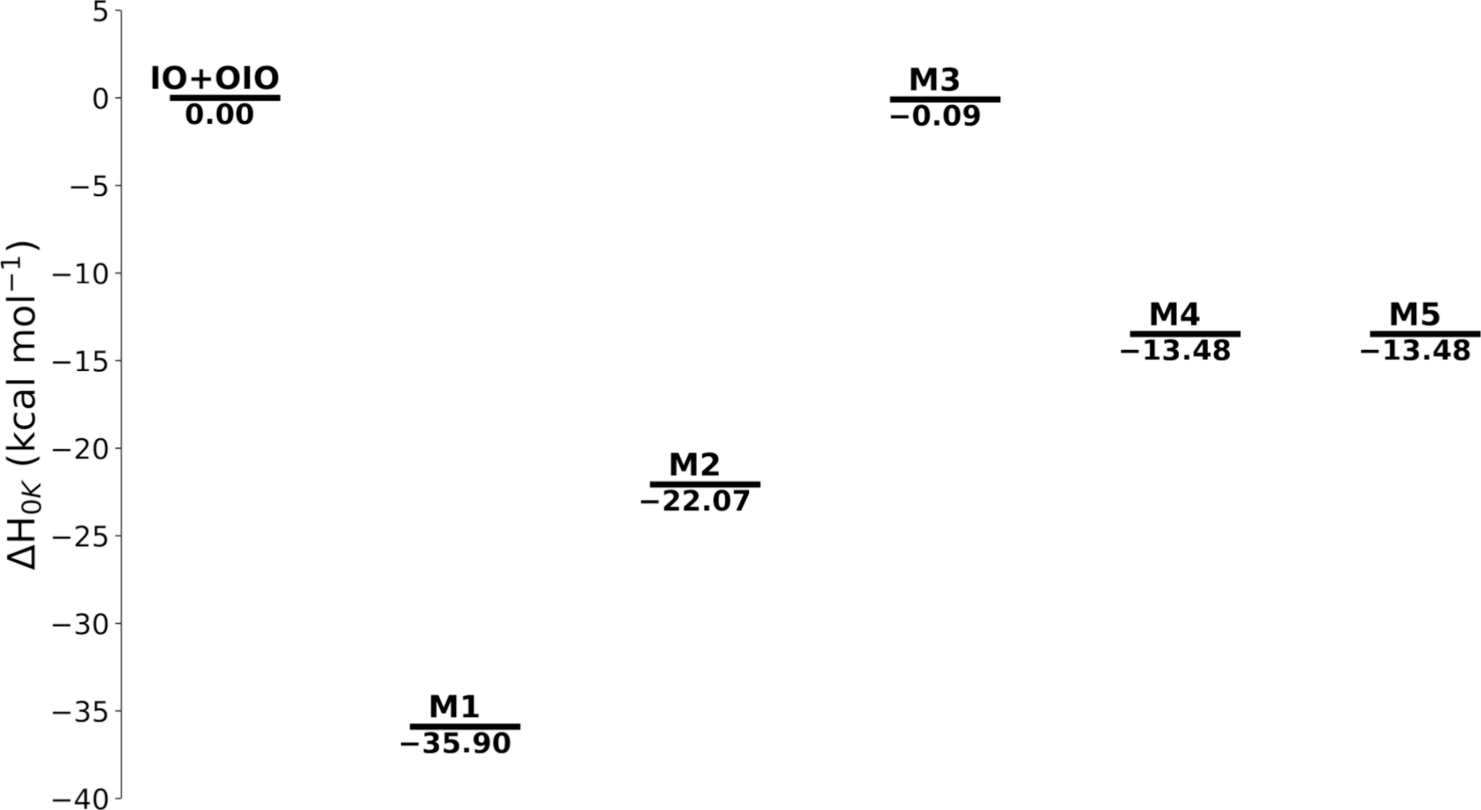
Energies of the I_2_O_3_ isomers from this study, in kcal mol^–1^, shown relative to the reactants.

The final energy reported by Kaltsoyannis and Plane was −39.75 kcal mol^–1^, nearly 4 kcal mol^–1^ different from the present research. Kaltsoyannis and Plane obtained their result using CCSD­(T) energies:

4 $$E\left(\mathrm{total}\right)=E\left(I_{2}O_{3}\right)-E\left(\mathrm{OIO}+\mathrm{IO}\right)+\delta_{\mathrm{ZPVE}}+\delta_{\mathrm{soc}}$$

where δ_ZPVE_ is a zero-point energy correction and δ_soc_ is a spin–orbit coupling correction.

The possibility of additional I_2_O_3_ isomers undergoing rearrangement to the global minimum is an intriguing consideration. The M2 and M3 isomers do not appear to have a straightforward pathway for rearrangement. However, the M4 and M5 isomers may be able to rearrange to the global minimum. Since the M4 and M5 isomers are isoenergetic and share very similar geometric parameters, the M5 isomer will be the focus of the following discussion. Notably, the M5 isomer also appears on the Cl_2_O_3_ potential energy surface reported by Zhu and Lin.^154^ Their surface is highly detailed, connecting six minima through 10 different transition states. On this surface, the Cl_2_O_3_ analog of the M5 isomer presented here can be connected to four different transition states. Two of those are particularly relevant: one connects the Cl_2_O_3_ analogs of the M1 and M5 isomers from this study, while the other connects the M5 analog to its reactants, ClO and OClO. Based on this, it may be possible for the I_2_O_3_ M5 isomer to undergo rearrangement to the M1 global minimum. However, further investigation into the transition states connecting the minima identified here would be needed to confirm this.

### Vibrational Frequencies

The vibrational frequencies of I_2_O_3_ have not been reported experimentally. The only available theoretical values are from Kaltsoyannis and Plane, calculated at the B3LYP/AVTZ level of theory.^89^ However, their computations were performed in C_1_ symmetry and did not include infrared intensity predictions. Table 3 presents anharmonic and harmonic vibrational frequencies computed in this study with the CCSD­(T)/ACVTZ, CCSD­(T)/AVTZ, and B3LYP/AVTZ methods, alongside the previously reported B3LYP/AVTZ values from Kaltsoyannis and Plane. Harmonic vibrational frequencies of IO, OIO, and the additional I_2_O_3_ isomers are shown in the Supporting Information.

**Table 3:** Vibrational Frequencies (cm^–1^), Infrared Intensities (km mol^–1^), and Symmetries of the Vibrational Modes of I_2_O_3_ Computed in This Study^a^

	CCSD(T)/ACVTZ**	CCSD(T)/ACVTZ	CCSD(T)/AVTZ	B3LYP/AVTZ	B3LYP/AVTZ*
ω_1_	34 (6, a″)	35 (6, a″)	32 (6, a″)	25 (5, a″)	43.4 (a)
ω_2_	97 (5, a′)	97 (5, a′)	96 (5, a′)	85 (4, a′)	88.2 (a)
ω_3_	239 (7, a″)	242 (7, a″)	236 (8, a″)	225 (7, a″)	236.8 (a)
ω_4_	256 (24, a′)	258 (24, a′)	253 (24, a′)	246 (24, a′)	254.6 (a)
ω_5_	301 (15, a′)	302 (15, a′)	294 (17, a′)	277 (16, a′)	297.5 (a)
ω_6_	467 (26, a′)	464 (27, a′)	466 (26, a′)	433 (30, a′)	442.5 (a)
ω_7_	638 (142, a′)	651 (156, a′)	653 (153, a′)	608 (102, a′)	625.7 (a)
ω_8_	861 (25, a′)	871 (25, a′)	872 (25, a′)	851 (35, a′)	872.7 (a)
ω_9_	890 (79, a″)	900 (83, a″)	902 (82, a″)	875 (92, a″)	901.2 (a)

a Unless otherwise noted, the vibrational frequencies were obtained using a harmonic approximation. The values for CCSD­(T)/ACVTZ**, noted with a double asterisk, were obtained using VPT2. The values for B3LYP/AVTZ*, noted with an asterisk, are from Kaltsoyannis and Plane.^89^

In Table 3, the largest difference between the anharmonic and harmonic frequencies is 13 cm^–1^. The largest difference between the anharmonic and harmonic intensities is only 6 km mol^–1^. Given the small differences between the anharmonic and harmonic results, the harmonic frequencies appear to be sufficient.

Discrepancies exist between the B3LYP harmonic vibrational frequencies obtained by Kaltsoyannis and Plane and those predicted in this study. Specifically, the B3LYP frequencies reported by Kaltsoyannis and Plane are slightly higher, ranging from 3 to 25 cm^–1^ higher.^89^ These B3LYP differences can likely be attributed to two factors. First, Kaltsoyannis and Plane employed a small all-electron basis set for iodine, whereas the present study utilized a 28-electron ECP with a better basis set. Second, their calculations were performed using the Amsterdam Density Functional (ADF) code, which employs Slater-type orbitals, in contrast to the Gaussian-type orbitals used in the codes for this study.

As with the geometric parameters, various method and basis set combinations were tested to evaluate their performance in predicting harmonic vibrational frequencies compared to CCSD­(T). These results are tabulated in the Supporting Information. Across all the methods, PW91 performs the worst due to consistently producing an imaginary vibrational mode for the global minimum. Similar to the findings for geometric parameters, B3LYP yields values closest to those of CCSD­(T). For double-ζ-quality basis sets, B3LYP outperforms MP2. However, with triple-ζ-quality basis sets, MP2 outperforms B3LYP for the lower frequency modes (ω_1–7_). For the higher frequency modes (ω_8_ and ω_9_), MP2 tends to overestimates the values, whereas B3LYP produces results closer to CCSD­(T).

From our results, mean absolute errors (MAE), root mean squared errors (RMSE), and maximum errors were determined for the harmonic vibrational frequencies to see how each method performed in comparison to CCSD­(T). When computing these metrics, imaginary ω_1_ values were omitted. These values are shown in Table 4. Similar to the geometric parameters, B3LYP is able to predict values closest to CCSD­(T). Surprisingly, MP2 performs the worst when considering these statistical metrics. This is likely due to the overestimation of the higher frequency modes mentioned above.

**Table 4:** Performance of Different Methods in Predicting Harmonic Vibrational Frequencies (cm^–1^) Relative to CCSD­(T) as Shown by Mean Absolute Error, Root-Mean-Square Error, and Maximum Error

	MAE	RMSE	Max. Error
MP2	33	59	195
CCSD	23	27	58
B3LYP	13	16	44
M06-2X	33	40	77
PW91	36	42	99
ωB97X-D	20	27	82

## Conclusions

The study focuses on the lowest energy I_2_O_3_ isomer, referred to as M1, and evaluates the performace of various computational methods and DFT functionals in comparison to CCSD­(T). To our knowledge, additional I_2_O_3_ structures are discovered here for the first time. Some discrepancies in the geometric parameters of the M1 isomer are observed when comparing this study’s results to three previous studies.^89,90,93^ These differences are likely due to three key factors. First, one study employed ADF which uses Slater-type orbitals and allows relativistic effects to be incorporated when using DFT.^89^ Second, the same study utilized an all-electron basis set for iodine atoms, whereas all other studies, including this one, used an ECP for iodine. Third, different studies employed varying ECPs; one used a 48-electron ECP, while this study and another used 28-electron ECPs.

When computing the energy of the M1 isomer relative to the reactants (IO and OIO), it was found that at the CCSD­(T)/CBS level of theory, the system lies −35.90 kcal mol^–1^ lower in energy with the inclusion of additional energy corrections. This is nearly 4 kcal mol^–1^ higher than the previously reported theoretical value of −39.75 kcal mol^–1^.^89^

When methods were standardized using consistent basis set and ECP combinations, B3LYP and MP2 were found to perform best in predicting geometric parameters relative to CCSD­(T). Of these, B3LYP slightly outperformed MP2, although MP2 remains a strong choice for avoiding DFT. Conversely, PW91 and CCSD showed the poorest performance.

In predicting harmonic vibrational frequencies, PW91 consistently produced an imaginary vibrational mode across all six basis set combinations. Similar to the geometry predictions, B3LYP performed the best overall. Specifically, B3LYP excelled when using double-ζ-quality basis sets, whereas MP2 performed better with triple-ζ basis sets for the lower-frequency modes (ω_1–7_). For higher-frequency modes (ω_8_ and ω_9_), MP2 tended to overestimate frequencies, while B3LYP produced results closer to CCSD­(T).

Aside from B3LYP, the DFT methods that were included in this study were chosen because they are often mentioned in the literature to accurately predict equilibrium geometries of atmospheric clusters.^3,159^ B3LYP was found to not only be the best performing functional, but best performing method, when considering the values presented in Tables 1 and 4 relative to CCSD­(T). As there are currently no experimental results for the structure of I_2_O_3_ and given the renewed interest in these types of iodine species, B3LYP appears to be the best choice for future studies if CCSD­(T) becomes too computationally expensive as the system size grows larger.

## Supplementary Material



## Notes

Geometric parameters, harmonic vibrational frequencies, and focal point tables of the reactants (IO and OIO) and I_2_O_3_ structures (PDF)
