# Supplementary material for: The Remarkable I2O3 Molecule: A New View from Theory
Source: J Phys Chem A. 2025 Aug 4;129(32):7507–16. doi: 10.1021/acs.jpca.5c04243 (PMC12359116; doi:10.1021/acs.jpca.5c04243)
Supplement: Supplementary file 1 [file jp5c04243_si_001.pdf]

Supplemental Information: The Remarkable I_2O_3 Molecule: A New View from Theory

Carson L. Tang, Justin M. Turney, Henry F. Schaefer III

Center for Computational Quantum Chemistry, University of Georgia

Athens, GA, USA, 30602

DZ	CCSD(T)	CCSD	MP2	B3LYP	M06-2X	PW91	ωB97X-D
$r_{(I_1-O_1)}$	2.059	2.044	2.050	2.046	2.023	2.052	2.034
$r_{(O_1-I_2)}$	2.040	2.002	2.059	2.044	1.982	2.087	2.006
$r_{(I_2-O_2)}$	1.826	1.811	1.791	1.823	1.798	1.843	1.801
$\alpha_{(I_1-O_1-I_2)}$	117.2	118.9	115.5	120.0	118.0	118.4	120.8
$\alpha_{(O_1-I_2-O_2)}$	102.7	102.5	103.0	103.5	101.6	104.0	102.6
$\alpha_{(O_2-I_2-O_3)}$	110.8	110.2	110.6	110.0	109.6	110.1	109.2
$\tau_{(I_1-O_1-I_2-O_2)}$	57.5	57.2	57.6	57.4	56.5	57.7	56.7

Table S1: Geometric parameters of the M1 structure of I_2O_3 computed here at various levels of theory, but all using the cc-pVDZ(-PP) basis set. All results reported here are from the present research. Bond lengths are shown in Å while bond angles and dihedrals are shown in degrees.

DZ	CCSD(T)	δCCSD	δMP2	δB3LYP	δM06-2X	δPW91	$\delta\omega$B97X-D
$r_{(I_1-O_1)}$	2.059	–0.015	–0.009	–0.013	–0.036	–0.007	–0.025
$r_{(O_1-I_2)}$	2.040	–0.038	0.019	0.004	–0.058	0.047	–0.034
$r_{(I_2-O_2)}$	1.826	–0.015	–0.035	–0.003	–0.028	0.017	–0.025
$\alpha_{(I_1-O_1-I_2)}$	117.2	1.7	–1.7	2.8	0.8	1.2	3.6
$\alpha_{(O_1-I_2-O_2)}$	102.7	–0.2	0.3	0.8	–1.1	1.3	–0.1
$\alpha_{(O_2-I_2-O_3)}$	110.8	–0.3	0.1	–0.1	–1.0	0.2	–0.8
$\tau_{(I_1-O_1-I_2-O_2)}$	57.5	–0.6	–0.2	–0.8	–1.2	–0.7	–1.6

Table S2: Geometric parameters of the M1 structure of I_2O_3 computed here at various levels of theory, but all using the cc-pVDZ(-PP) basis set. All results reported here are from the present research and are relative to CCSD(T). Bond lengths are shown in Å while bond angles and dihedrals are shown in degrees.

AVDZ	CCSD(T)	CCSD	MP2	B3LYP	M06-2X	PW91	ωB97X-D
$r_{(I_1-O_1)}$	2.043	2.030	2.040	2.039	2.020	2.043	2.029
$r_{(O_1-I_2)}$	2.018	1.983	2.045	2.037	1.972	2.085	1.997
$r_{(I_2-O_2)}$	1.824	1.810	1.797	1.822	1.797	1.841	1.801
$\alpha_{(I_1-O_1-I_2)}$	115.5	117.1	113.7	119.0	116.3	117.2	119.5
$\alpha_{(O_1-I_2-O_2)}$	102.7	102.5	103.1	103.6	101.7	103.9	102.6
$\alpha_{(O_2-I_2-O_3)}$	109.6	109.2	109.5	109.3	108.8	109.4	108.5
$\tau_{(I_1-O_1-I_2-O_2)}$	56.9	56.6	57.0	57.0	56.2	57.2	56.3

Table S3: Geometric parameters of the M1 structure of I_2O_3 computed here at various levels of theory, but all using the aug-cc-pVDZ(-PP) basis set. All results reported here are from the present research. Bond lengths are shown in Å while bond angles and dihedrals are shown in degrees.

AVDZ	CCSD(T)	δCCSD	δMP2	δB3LYP	δM06-2X	δPW91	$\delta\omega$B97X-D
$r_{(I_1-O_1)}$	2.043	–0.013	–0.003	–0.004	–0.023	0.000	–0.014
$r_{(O_1-I_2)}$	2.018	–0.035	0.027	0.019	–0.046	0.067	–0.021
$r_{(I_2-O_2)}$	1.824	–0.014	–0.027	–0.002	–0.027	0.017	–0.023
$\alpha_{(I_1-O_1-I_2)}$	115.5	1.6	–1.8	3.5	0.8	1.7	4.0
$\alpha_{(O_1-I_2-O_2)}$	102.7	–0.2	0.4	0.9	–1.0	1.2	–0.1
$\alpha_{(O_2-I_2-O_3)}$	109.6	–0.3	0.1	0.1	–0.7	0.3	–0.6
$\tau_{(I_1-O_1-I_2-O_2)}$	56.9	–0.4	–0.1	–0.3	–0.8	–0.2	–1.1

Table S4: Geometric parameters of the M1 structure of I_2O_3 computed here at various levels of theory, but all using the aug-cc-pVDZ(-PP) basis set. All results reported here are from the present research and are relative to CCSD(T). Bond lengths are shown in Å while bond angles and dihedrals are shown in degrees.

ACVDZ	CCSD(T)	CCSD	MP2	B3LYP	M06-2X	PW91	ωB97X-D
$r_{(I_1-O_1)}$	2.034	2.021	2.031	2.030	2.012	2.036	2.021
$r_{(O_1-I_2)}$	2.008	1.971	2.036	2.016	1.953	2.064	1.978
$r_{(I_2-O_2)}$	1.809	1.792	1.787	1.800	1.776	1.820	1.780
$\alpha_{(I_1-O_1-I_2)}$	115.5	117.2	113.3	119.3	116.7	117.2	120.3
$\alpha_{(O_1-I_2-O_2)}$	102.7	102.5	103.1	103.5	101.7	103.8	102.5
$\alpha_{(O_2-I_2-O_3)}$	109.1	108.7	108.8	108.8	108.4	108.8	108.1
$\tau_{(I_1-O_1-I_2-O_2)}$	56.6	56.3	56.6	56.7	56.0	56.9	56.1

Table S5: Geometric parameters of the M1 structure of I_2O_3 computed here at various levels of theory, but all using the aug-cc-pwCVDZ(-PP) basis set. All results reported here are from the present research. Bond lengths are shown in Å while bond angles and dihedrals are shown in degrees.

ACVDZ	CCSD(T)	δCCSD	δMP2	δB3LYP	δM06-2X	δPW91	$\delta\omega$B97X-D
$r_{(I_1-O_1)}$	2.034	–0.013	–0.003	–0.004	–0.022	0.002	–0.013
$r_{(O_1-I_2)}$	2.008	–0.037	0.028	0.008	–0.055	0.056	–0.030
$r_{(I_2-O_2)}$	1.809	–0.017	–0.022	–0.009	–0.033	0.011	–0.029
$\alpha_{(I_1-O_1-I_2)}$	115.5	1.7	–2.2	3.8	1.2	1.7	4.8
$\alpha_{(O_1-I_2-O_2)}$	102.7	–0.2	0.4	0.8	–1.0	1.1	–0.2
$\alpha_{(O_2-I_2-O_3)}$	109.1	–0.3	0.0	0.1	–0.6	0.3	–0.5
$\tau_{(I_1-O_1-I_2-O_2)}$	56.6	–0.4	–0.3	–0.3	–0.7	–0.3	–1.0

Table S6: Geometric parameters of the M1 structure of I_2O_3 computed here at various levels of theory, but all using the aug-cc-pwCVDZ(-PP) basis set. All results reported here are from the present research and are relative to CCSD(T). Bond lengths are shown in Å while bond angles and dihedrals are shown in degrees.

TZ	CCSD(T)	CCSD	MP2	B3LYP	M06-2X	PW91	ωB97X-D
$r_{(I_1-O_1)}$	2.012	1.985	1.997	2.019	1.999	2.026	2.005
$r_{(O_1-I_2)}$	1.960	1.912	1.965	1.991	1.930	2.034	1.956
$r_{(I_2-O_2)}$	1.778	1.751	1.759	1.789	1.765	1.808	1.769
$\alpha_{(I_1-O_1-I_2)}$	116.4	119.1	114.1	120.0	118.7	117.3	121.1
$\alpha_{(O_1-I_2-O_2)}$	102.0	101.8	102.2	103.0	101.4	103.2	102.2
$\alpha_{(O_2-I_2-O_3)}$	108.3	107.2	107.0	108.1	107.9	108.1	107.5
$\tau_{(I_1-O_1-I_2-O_2)}$	56.0	55.3	55.3	56.2	55.6	56.3	55.6

Table S7: Geometric parameters of the M1 structure of I_2O_3 computed here at various levels of theory, but all using the cc-pVTZ(-PP) basis set. All results reported here are from the present research. Bond lengths are shown in Å while bond angles and dihedrals are shown in degrees.

TZ	CCSD(T)	δCCSD	δMP2	δB3LYP	δM06-2X	δPW91	$\delta\omega$B97X-D
$r_{(I_1-O_1)}$	2.012	–0.027	–0.015	0.007	–0.013	0.014	–0.007
$r_{(O_1-I_2)}$	1.960	–0.048	0.005	0.031	–0.030	0.074	–0.004
$r_{(I_2-O_2)}$	1.778	–0.027	–0.019	0.011	–0.013	0.030	–0.009
$\alpha_{(I_1-O_1-I_2)}$	116.4	2.7	–2.3	3.6	2.3	0.9	4.7
$\alpha_{(O_1-I_2-O_2)}$	102.0	–0.2	0.2	1.0	–0.6	1.2	0.2
$\alpha_{(O_2-I_2-O_3)}$	108.3	–0.7	–0.7	0.2	–0.4	0.3	–0.4
$\tau_{(I_1-O_1-I_2-O_2)}$	56.0	–1.1	–1.3	–0.2	–0.4	–0.2	–0.8

Table S8: Geometric parameters of the M1 structure of I_2O_3 computed here at various levels of theory, but all using the cc-pVTZ(-PP) basis set. All results reported here are from the present research and are relative to CCSD(T). Bond lengths are shown in Å while bond angles and dihedrals are shown in degrees.

AVTZ	CCSD(T)	CCSD	MP2	B3LYP	M06-2X	PW91	ωB97X-D
$r_{(I_1-O_1)}$	2.006	1.985	1.997	2.018	2.000	2.025	2.005
$r_{(O_1-I_2)}$	1.955	1.912	1.965	1.989	1.928	2.035	1.954
$r_{(I_2-O_2)}$	1.781	1.751	1.759	1.790	1.767	1.809	1.771
$\alpha_{(I_1-O_1-I_2)}$	116.3	119.1	114.1	120.6	119.0	117.8	121.6
$\alpha_{(O_1-I_2-O_2)}$	101.8	101.8	102.2	103.2	101.5	103.4	102.3
$\alpha_{(O_2-I_2-O_3)}$	108.0	107.2	107.0	108.0	107.7	108.0	107.4
$\tau_{(I_1-O_1-I_2-O_2)}$	55.8	55.3	55.3	56.2	55.5	56.2	55.6

Table S9: Geometric parameters of the M1 structure of I_2O_3 computed here at various levels of theory, but all using the aug-cc-pVTZ(-PP) basis set. All results reported here are from the present research. Bond lengths are shown in Å while bond angles and dihedrals are shown in degrees.

AVTZ	CCSD(T)	δCCSD	δMP2	δB3LYP	δM06-2X	δPW91	$\delta\omega$B97X-D
$r_{(I_1-O_1)}$	2.006	–0.021	–0.009	0.012	–0.006	0.019	–0.001
$r_{(O_1-I_2)}$	1.955	–0.043	0.010	0.034	–0.027	0.080	–0.001
$r_{(I_2-O_2)}$	1.781	–0.030	–0.022	0.009	–0.014	0.028	–0.010
$\alpha_{(I_1-O_1-I_2)}$	116.3	2.8	–2.2	4.3	2.7	1.5	5.3
$\alpha_{(O_1-I_2-O_2)}$	101.8	0.0	0.4	1.4	–0.3	1.6	0.5
$\alpha_{(O_2-I_2-O_3)}$	108.0	–0.5	–0.5	0.4	–0.3	0.4	–0.2
$\tau_{(I_1-O_1-I_2-O_2)}$	55.8	–0.8	–1.0	0.0	–0.3	0.0	–0.6

Table S10: Geometric parameters of the M1 structure of I_2O_3 computed here at various levels of theory, but all using the aug-cc-pVTZ(-PP) basis set. All results reported here are from the present research and are relative to CCSD(T). Bond lengths are shown in Å while bond angles and dihedrals are shown in degrees.

ACVTZ	CCSD(T)	CCSD	MP2	B3LYP	M06-2X	PW91	ωB97X-D
$r_{(I_1-O_1)}$	2.000	1.985	1.997	2.010	1.993	2.018	1.999
$r_{(O_1-I_2)}$	1.943	1.912	1.965	1.966	1.909	2.009	1.934
$r_{(I_2-O_2)}$	1.767	1.751	1.759	1.773	1.750	1.791	1.754
$\alpha_{(I_1-O_1-I_2)}$	116.7	119.1	114.1	121.2	119.7	118.2	122.8
$\alpha_{(O_1-I_2-O_2)}$	102.0	101.8	102.2	103.0	101.5	103.3	102.2
$\alpha_{(O_2-I_2-O_3)}$	107.3	107.2	107.0	107.2	107.0	107.1	106.6
$\tau_{(I_1-O_1-I_2-O_2)}$	55.4	55.3	55.3	55.7	55.1	55.7	55.1

Table S11: Geometric parameters of the M1 structure of I_2O_3 computed here at various levels of theory, but all using the aug-cc-pwCVTZ(-PP) basis set. All results reported here are from the present research. Bond lengths are shown in Å while bond angles and dihedrals are shown in degrees.

ACVTZ	CCSD(T)	δCCSD	δMP2	δB3LYP	δM06-2X	δPW91	$\delta\omega$B97X-D
$r_{(I_1-O_1)}$	2.000	–0.015	–0.003	0.010	–0.007	0.018	–0.001
$r_{(O_1-I_2)}$	1.943	–0.031	0.022	0.023	–0.034	0.066	–0.009
$r_{(I_2-O_2)}$	1.767	–0.016	–0.008	0.006	–0.017	0.024	–0.013
$\alpha_{(I_1-O_1-I_2)}$	116.7	2.4	–2.6	4.5	3.0	1.5	6.1
$\alpha_{(O_1-I_2-O_2)}$	102.0	–0.2	0.2	1.0	–0.5	1.3	0.2
$\alpha_{(O_2-I_2-O_3)}$	107.3	–0.1	–0.1	0.3	–0.3	0.3	–0.3
$\tau_{(I_1-O_1-I_2-O_2)}$	55.4	–0.1	–0.3	–0.1	–0.3	–0.2	–0.7

Table S12: Geometric parameters of the M1 structure of I_2O_3 computed here at various levels of theory, but all using the aug-cc-pwCVTZ(-PP) basis set. All results reported here are from the present research and are relative to CCSD(T). Bond lengths are shown in Å while bond angles and dihedrals are shown in degrees.

DZ	CCSD(T)	CCSD	MP2	B3LYP	M06-2X	PW91	ωB97X-D
ω_1	$9i$	19	15	$9i$	35	$23i$	12
ω_2	87	92	90	80	95	70	89
ω_3	217	236	220	213	245	194	234
ω_4	230	246	246	231	257	214	249
ω_5	270	284	300	272	290	257	293
ω_6	416	450	398	425	490	399	445
ω_7	603	657	599	595	680	550	634
ω_8	792	817	987	812	865	778	874
ω_9	841	865	996	847	906	814	906
AVDZ	CCSD(T)	CCSD	MP2	B3LYP	M06-2X	PW91	ωB97X-D
ω_1	14	25	19	12	42	$24i$	17
ω_2	93	97	93	84	101	74	93
ω_3	214	232	212	208	242	190	229
ω_4	233	248	243	231	255	213	247
ω_5	271	285	294	272	291	256	291
ω_6	447	477	415	431	505	400	459
ω_7	619	668	603	590	675	548	629
ω_8	808	833	963	813	868	781	872
ω_9	840	865	972	837	897	805	893
ACVDZ	CCSD(T)	CCSD	MP2	B3LYP	M06-2X	PW91	ωB97X-D
ω_1	21	30	24	14	48	$24i$	22
ω_2	94	98	94	85	102	76	93
ω_3	218	237	213	215	251	196	236
ω_4	237	253	242	238	262	220	254
ω_5	276	291	291	280	300	264	300
ω_6	441	472	410	429	500	401	455
ω_7	613	663	596	589	676	544	632
ω_8	799	829	933	815	871	781	870
ω_9	831	860	939	842	901	807	894

Table S13: Harmonic vibrational frequencies (cm^{-1}) computed in this study for the global minimum M1 isomer with double-zeta quality basis sets. Imaginary vibrational modes are shown in red text. All results reported in this table are from the present research.

TZ	CCSD(T)	CCSD	MP2	B3LYP	M06-2X	PW91	ωB97X-D
ω_1	38	44	41	23	36	22*i*	34
ω_2	98	100	99	85	100	77	93
ω_3	243	261	239	229	264	210	250
ω_4	258	272	261	249	273	231	266
ω_5	300	315	312	294	316	278	314
ω_6	464	488	447	438	500	416	466
ω_7	647	702	634	609	703	556	670
ω_8	878	908	983	858	914	821	909
ω_9	914	943	989	885	945	849	935
AVTZ	CCSD(T)	CCSD	MP2	B3LYP	M06-2X	PW91	ωB97X-D
ω_1	32	39	35	25	36	20*i*	35
ω_2	96	99	97	85	99	76	93
ω_3	236	254	231	225	259	205	246
ω_4	253	268	256	246	270	229	264
ω_5	294	309	304	277	313	275	311
ω_6	466	490	447	433	496	410	462
ω_7	653	710	635	609	702	554	669
ω_8	872	903	975	857	907	814	901
ω_9	902	933	975	875	934	838	923
ACVTZ	CCSD(T)	CCSD	MP2	B3LYP	M06-2X	PW91	ωB97X-D
ω_1	35	41	37	28	31	17*i*	38
ω_2	97	99	98	85	99	78	92
ω_3	242	261	235	233	267	213	253
ω_4	258	274	257	254	276	237	271
ω_5	302	318	307	302	323	285	321
ω_6	464	488	447	435	495	415	457
ω_7	651	709	631	617	710	558	675
ω_8	871	908	952	864	919	826	912
ω_9	900	936	954	888	944	850	934

Table S14: Harmonic vibrational frequencies (cm^{-1}) computed in this study for the global minimum M1 isomer with triple-zeta quality basis sets. Imaginary vibrational modes are shown in red text. All results reported in this table are from the present research.

IO		OIO		M2		M3		M4		M5	
$r_{(I-O)}$	1.877	$r_{(O-I)}$	1.804	$r_{(I_1-O_1)}$	2.024	$r_{(I_1-I_2)}$	2.714	$r_{(I_1-O_1)}$	2.012	$r_{(I_1-O_1)}$	2.012
		$\alpha_{(O-I-O)}$	109.9	$r_{(O_1-O_2)}$	1.414	$r_{(I_2-O_1)}$	1.765	$r_{(O_1-O_2)}$	1.446	$r_{(O_1-O_2)}$	1.454
				$\alpha_{(I_1-O_1-O_2)}$	111.1	$r_{(O_1-O_2)}$	2.951	$r_{(O_2-I_2)}$	2.005	$r_{(O_2-I_2)}$	1.999
				$\alpha_{(O_1-O_2-O_3)}$	108.4	$\alpha_{(I_1-I_2-O_1)}$	105.1	$r_{(I_2-O_3)}$	1.812	$r_{(I_2-O_3)}$	1.814
				$\tau_{(I_1-O_1-O_2-O_3)}$	−72.5	$\alpha_{(O_1-I_2-O_2)}$	113.5	$\alpha_{(I_1-O_1-O_2)}$	110.2	$\alpha_{(I_1-O_1-O_2)}$	109.9
						$\tau_{(I_1-I_2-O_1-O_2)}$	−114.3	$\alpha_{(O_1-O_2-I_2)}$	110.5	$\alpha_{(O_1-O_2-I_2)}$	110.4
								$\alpha_{(O_2-I_2-O_3)}$	106.0	$\alpha_{(O_2-I_2-O_3)}$	106.4
								$\tau_{(I_1-O_1-O_2-I_2)}$	−89.7	$\tau_{(I_1-O_1-O_2-I_2)}$	−99.0
								$\tau_{(O_1-O_2-I_2-O_3)}$	−75.0	$\tau_{(O_1-O_2-I_2-O_3)}$	89.8

Table S15: Geometric parameters of IO, OIO, and the higher energy I_2O_3 isomers computed in this research at the CCSD(T)/ACVTZ level of theory. Bond lengths are shown in angstrom while bond angles and dihedrals are shown in degrees.

Basis Set	RHF	$+\delta$MP2	$+\delta$CCSD	$+\delta$(T)	Net
DZ	–19.79	+0.35	–3.09	+2.60	–19.93
TZ	–8.47	–4.42	–3.45	+2.41	–13.93
QZ	–7.53	–3.59	–3.83	+2.41	–12.54
5Z	–7.37	–3.73	–3.79	+2.39	–12.50
CBS	[–7.35]	[–2.98]	[–4.10]	[+2.40]	[–12.02]
$\Delta_f H_0 = -12.02 + 0.93 + 0.42 + 1.59 - 12.99 + 1.00 = -22.07 \pm 2.23$ kcal mol^{-1}					

Table S16: Incremental focal point table for the M2 isomer relative to the reactants. The final energy is obtained by the following: $\Delta_f H_0 = \Delta E_{\text{CCSD(T)/CBS}} + \delta_{\text{T}} + \delta_{\text{(Q)}} + \delta_{\text{ZPVE}} + \delta_{\text{Rel.}} + \delta_{SO}$

Basis Set	RHF	$+\delta$MP2	$+\delta$CCSD	$+\delta$(T)	Net
DZ	+17.97	–22.15	+9.87	–3.46	+2.23
TZ	+4.57	–25.74	+11.60	–3.67	–13.24
QZ	+4.01	–27.57	+11.50	–3.85	–15.91
5Z	+3.93	–28.32	+11.48	–3.93	–16.83
CBS	[+3.93]	[–28.91]	[+11.42]	[–3.98]	[–17.54]
$\Delta_f H_0 = -17.54 + 0.93 - 1.01 + 1.68 + 15.85 + 1.00 = -0.09 \pm 1.06$ kcal mol^{-1}					

Table S17: Incremental focal point table for the M3 isomer relative to the reactants. The final energy is obtained by the following: $\Delta_f H_0 = \Delta E_{\text{CCSD(T)/CBS}} + \delta_{\text{T}} + \delta_{\text{(Q)}} + \delta_{\text{ZPVE}} + \delta_{\text{Rel.}} + \delta_{SO}$

Basis Set	RHF	$+\delta$MP2	$+\delta$CCSD	$+\delta$(T)	Net
DZ	+0.48	–15.08	+2.81	–0.25	–12.04
TZ	+6.00	–19.30	+3.83	–0.68	–10.15
QZ	+6.29	–18.98	+3.77	–0.77	–9.69
5Z	+6.38	–19.18	[+24.44]	[–0.42]	[+11.23]
CBS	[+6.42]	[–18.75]	[+3.72]	[–0.83]	[–9.44]
$\Delta_f H_0 = -9.44 + 0.80 + -0.37 + 1.06 - 5.53 + 1.00 = -13.48 \pm 1.43$ kcal mol^{-1}					

Table S18: Incremental focal point table for the M4 isomer relative to the reactants. The final energy is obtained by the following: $\Delta_f H_0 = \Delta E_{\text{CCSD(T)/CBS}} + \delta_{\text{T}} + \delta_{\text{(Q)}} + \delta_{\text{ZPVE}} + \delta_{\text{Rel.}} + \delta_{SO}$

Basis Set	RHF	+δMP2	+δCCSD	+δ(T)	Net
DZ	–0.10	–14.33	+2.43	–0.09	–12.08
TZ	+5.36	–18.41	+3.37	–0.47	–10.15
QZ	+5.66	–18.07	+3.30	–0.54	–9.64
5Z	+5.76	[+6.56]	[–0.85]	[–0.19]	[+11.28]
CBS	[+5.79]	[–17.82]	[+3.25]	[–0.59]	[–9.37]
$\Delta_f H_0 = -9.37 + 0.84 - 0.35 + 1.00 - 5.60 + 1.00 = -13.48 \pm 1.41$ kcal mol^{-1}					

Table S19: Incremental focal point table for the M5 isomer relative to the reactants. The final energy is obtained by the following: $\Delta_f H_0 = \Delta E_{\mathrm{CCSD(T)/CBS}} + \delta_{\mathrm{T}} + \delta_{(\mathrm{Q})} + \delta_{\mathrm{ZPVE}} + \delta_{\mathrm{Rel.}} + \delta_{SO}$

	IO	OIO	M2	M3	M4	M5
ω_1	672 (1, σ_g^+)	265 (15, a_1)	30 (0, a)	137 (1, e)	40 (1, a)	25 (5, a)
ω_2		792 (3, a_1)	124 (3, b)	137 (1, e)	70 (7, a)	61 (9, a)
ω_3		980 (3411, b_2)	170 (1, a)	166 (0, a_1)	143 (2, a)	164 (9, a)
ω_4			358 (0, b)	269 (17, e)	264 (8, a)	217 (4, a)
ω_5			500 (1, a)	269 (17, e)	327 (5, a)	325 (6, a)
ω_6			537 (1, b)	290 (93, a_1)	503 (17, a)	522 (17, a)
ω_7			582 (1, a)	833 (27, a_1)	564 (1, a)	568 (1, a)
ω_8			677 (14, b)	892 (53, e)	736 (12, a)	731 (11, a)
ω_9			841 (3, a)	892 (53, e)	804 (53, a)	799 (33, a)

Table S20: Harmonic vibrational frequencies (cm^{-1}), intensities (km mol^{-1}), and symmetries of the vibrational modes of IO, OIO, and additional I_2O_3 isomers computed at the CCSD(T)/ACVTZ level of theory.
